# Insights into the Evolutionary History of an Extinct South American Freshwater Snail Based on Historical DNA

**DOI:** 10.1371/journal.pone.0169191

**Published:** 2016-12-29

**Authors:** Roberto E. Vogler, Ariel A. Beltramino, Ellen E. Strong, Alejandra Rumi, Juana G. Peso

**Affiliations:** 1 Instituto de Biología Subtropical, Universidad Nacional de Misiones, CONICET, Posadas, Misiones, Argentina; 2 División Zoología Invertebrados, Facultad de Ciencias Naturales y Museo, Universidad Nacional de La Plata, CONICET, La Plata, Buenos Aires, Argentina; 3 Departamento de Biología, Facultad de Ciencias Exactas, Químicas y Naturales, Universidad Nacional de Misiones, CONICET, Posadas, Misiones, Argentina; 4 Smithsonian Institution, National Museum of Natural History, Washington, D.C., United States of America; University of California, UNITED STATES

## Abstract

Highly oxygenated freshwater habitats in the High Paraná River (Argentina–Paraguay) were home to highly endemic snails of the genus *Aylacostoma*, which face extinction owing to the impoundment of the Yacyretá Reservoir in the 1990s. Two species, *A*. *chloroticum* and *A*. *brunneum*, are currently included in an ongoing *ex situ* conservation programme, whereas *A*. *guaraniticum* and *A*. *stigmaticum* are presumed extinct. Consequently, the validity and affinities of the latter two have remained enigmatic. Here, we provide the first molecular data on the extinct *A*. *stigmaticum* by means of historical DNA analysis. We describe patterns of molecular evolution based on partial sequences of the mitochondrial 12S ribosomal RNA gene from the extinct species and from those being bred within the *ex situ* programme. We further use this gene to derive a secondary structure model, to examine the specific status of *A*. *stigmaticum* and to explore the evolutionary history of these snails. The secondary structure model based on *A*. *stigmaticum* revealed that most polymorphic sites are located in unpaired regions. Our results support the view that the mitochondrial 12S region is an efficient marker for the discrimination of species, and the extinct *A*. *stigmaticum* is recognized here as a distinct evolutionary genetic species. Molecular phylogenetic analyses revealed a sister group relationship between *A*. *chloroticum* and *A*. *brunneum*, and estimated divergence times suggest that diversification of *Aylacostoma* in the High Paraná River might have started in the late Miocene via intra-basin speciation due to a past marine transgression. Finally, our findings reveal that DNA may be obtained from dried specimens at least 80 years after their collection, and confirms the feasibility of extracting historical DNA from museum collections for elucidating evolutionary patterns and processes in gastropods.

## Introduction

Freshwater gastropods account for a disproportionate number of documented molluscan extinctions, which are the highest recorded for any major taxonomic group [1–3]. Freshwater molluscs have experienced severe declines as a consequence of a variety of human-mediated impacts, with many species imperilled or facing extinction [2–8]. Known examples include rivers with species-rich assemblages of molluscs such as the Pleuroceridae from the Mobile River basin in the southeastern United States and the Rissooidea from the Mekong River in Southeast Asia, where extensive impoundment of mainstem rivers has resulted in the loss of many species [1–3]. An emblematic example of human-mediated extinction within the Mollusca can be found among South American freshwater snails of the genus *Aylacostoma* Spix, 1827 (Caenogastropoda: Thiaridae) [9,10]. The members of this endemic genus have been known since the 19^th^ century, but have received little attention since the original descriptions [10–13]. Nonetheless, some *Aylacostoma* species from the High Paraná River between Argentina and Paraguay have attracted the interest of researchers and conservation biologists owing to an impending extinction in response to human-mediated modifications to their habitat [9,10].

Historically, the highly oxygenated fresh waters near the Yacyretá–Apipé rapids in the High Paraná River were home to five *Aylacostoma* species: *Aylacostoma guaraniticum* (Hylton Scott, 1953), *Aylacostoma chloroticum* Hylton Scott, 1954, *Aylacostoma stigmaticum* Hylton Scott, 1954, and two others that have never been formally described [9,14–16]. The decline of this mollusc fauna began in the early 1990s in response to the impoundment of the Yacyretá Reservoir for a binational hydroelectric power plant between Argentina and Paraguay [10,13,16]. With the progressive filling of the reservoir, most *Aylacostoma* populations experienced drastic range reductions, with all species except *A*. *chloroticum* going extinct in the wild [13,16,17]. Conservation strategies were adopted during the initial filling stages in the 1990s through an ongoing *ex situ* conservation programme, known as the “*Aylacostoma* Project” hosted by the Universidad Nacional de Misiones (UNaM; Posadas, Argentina), in conjunction with the Museo Argentino de Ciencias Naturales (MACN; Buenos Aires, Argentina), and supported by the Entidad Binacional Yacyretá [18]. At present, only *A*. *chloroticum* and the recently described *Aylacostoma brunneum* Vogler & Peso, 2014 [13] are being maintained in captivity. The former species is restricted to only one known wild population in a small and fragile habitat under anthropogenic threat due to the construction of an artificial beach for human recreation. Wild populations of *A*. *brunneum* are presumed to have gone extinct after their habitats were flooded in 2011 during the final filling stage of the reservoir [13,16]. *Aylacostoma guaraniticum* and *A*. *stigmaticum* are not represented by any captive population and are presumed extinct [19,20].

Recent morphological and genetic studies investigating the taxonomic status and phylogeography of captive *Aylacostoma* populations from the High Paraná River, provided critical historical information for maintaining the integrity of evolutionarily lineages in the *ex situ* breeding program, as well as for future reintroductions and translocations [13,16]. However, owing to extinction, the validity and affinities of *A*. *guaraniticum* and *A*. *stigmaticum* have remained enigmatic, as the only material available for study in natural history collections dates back to the early 1930s, and is represented only by dried shells of the type series. Moreover, soft-part anatomy is poorly documented for *A*. *guaraniticum* [21], and is not available for *A*. *stigmaticum* whose radula was characterized for the first time from a dry syntype by Vogler [18].

In the present study, we provide the first molecular data for the extinct freshwater snail *A*. *stigmaticum* obtained from a dry syntype. Using information from the third domain of the mitochondrial 12S rRNA gene and a secondary structure model of this region, we assessed the evolutionary relationships among the extinct species and those successfully being bred in captivity. We further examined the validity of *A*. *stigmaticum* using two methods implemented for single locus data, and estimated divergence times in order to establish a timeframe for diversification. The goal of this study is to provide insights into the evolutionary history of *Aylacostoma* snails, to shed light on the possible scenarios involved in the diversification and evolution of this endemic genus in the High Paraná River.

## Material and Methods

### Samples and DNA extraction

Total genomic DNA of *A*. *stigmaticum* was obtained from a dry syntype housed in the malacological collection of the Museo de La Plata (MLP 10965; La Plata, Argentina) from which the radula was recovered by Vogler [18] following a modification of the non-destructive method described by Holznagel [22]. The shell was rinsed with deionized water, placed in a 10 ml tube, with 1.5 ml of NET buffer (10 mM Tris, 10 mM EDTA, 2% SDS) and 10 μl of proteinase K (20 mg/ml), and incubated at 37°C for seven days; an identical volume of proteinase K was added on the third day. After incubation, a gelatinous mass containing the radula was recovered from inside the shell. This mass was further incubated at 56°C with 500 μl of NET buffer and 10 μl of proteinase K for 24 hrs, eventually producing the clean radula. DNA was purified from the remaining solution by a threefold extraction, one with phenol and two with chloroform-isoamyl alcohol (24:1) followed by precipitation with cold isopropanol. DNA was then resuspended in 50 μl of 10 mM Tris/HCl. In addition, a genomic DNA collection of *Aylacostoma* specimens from the ongoing *ex situ* conservation programme at UNaM collected in the High Paraná River between 1994 and 2011 was made available at MLP by Vogler et al. [13]. We selected 13 samples from that collection belonging to *A*. *chloroticum*, *A*. *brunneum* and the outgroups *Doryssa* sp., *Pachychilus nigratus* (Poey, 1858), and *Pachychilus laevissimus* (Sowerby, 1825). Samples of *A*. *chloroticum* included representatives of both haplotype clades [16]. Collection information and GenBank accession numbers for the samples analysed is presented in Table 1.

**Table 1:** Collection information and GenBank accession numbers for the samples analysed herein.

Species	Locality	Coordinates	Year	*N*	GenBank accession nos.
*Aylacostoma stigmaticum*	Isla Ibicuy, PY*	27°17′56.76′′S; 56°3′28.44′′W**	1934	1	KU168372
*Aylacostoma brunneum*	Ita Cuá, PY	27°24′42.13′′S; 55°48′45.69′′W	2007	1	KU168374
	Río Beach, PY*	27°24′29.83′′S; 55°49′32.94′′W	2007	2	KU168373, KU168375
*Aylacostoma chloroticum*	Candelaria, AR	27°26′50.96′′S; 55°45′0.84′′W	2008	4	KU168376 –KU168379
			2011	2	KU168380, KU168381
	Río Beach, PY	27°24′29.83′′S; 55°49′32.94′′W	2007	1	KU168382
*Doryssa* sp.***	Venezuela	–	2010	1	KU168383
Pachychilus laevissimus***	Venezuela	–	2010	1	KU168384
Pachychilus nigratus***	Cuba	–	1997	1	KU168385

AR, Argentina; PY, Paraguay; *N*, number of specimens analysed.

*Type locality.

**The coordinates are approximate; only the locality name was reported in the original description and the precise location is uncertain.

***Outgroup species.

### PCR amplification and sequencing

Repeated attempts to amplify partial fragments of the mitochondrial cytochrome *c* oxidase subunit I (COI) and cytochrome *b* (cyt *b*) genes of *A*. *stigmaticum* following the methodology of Vogler et al. [13] were unsuccessful. Consequently, the mitochondrial 12S rRNA gene (hereafter 12S) was selected for further characterization based on previous research showing its potential utility in other gastropods (e.g. [23–25]). Partial sequences of the 12S gene were amplified with the 12SF (5′-AAC TCA AAG GAC TTG GCG GTG C-3′) and 12SR (5′-GTT TTT TTA CTT TCA AGT CCT CC-3′) primers [24]. PCR was performed in a total volume of 50 μl containing 30–50 ng of template DNA, 0.5 μM of each primer, 1X PCR buffer, 0.2 mM dNTPs, 1.5 mM MgCl_2_, and 1.5 U Platinum *Taq* polymerase (Invitrogen, Brazil). The thermocycling profile was 1 min at 94°C followed by 40 cycles of 30 s at 94°C, 30 s at 55°C, 1 min at 72°C. Amplifications were run on a T18 thermocycler (Ivema Desarrollos). Success of PCR reactions was verified by agarose gel electrophoresis. Owing to the co-amplification of nonspecific fragments, PCR products were purified from 1.5% (w/v) agarose gel through the use of a Zymoclean Gel DNA Recovery Kit (Zymo Research, Orange, California). After purification, both DNA strands were then directly cycle sequenced (Macrogen Inc, Seoul, Korea). The resulting sequences were trimmed to remove the primers, and the consensus sequences compared with reference sequences in GenBank through the use of the BLASTN algorithm [26] to rule out potential contamination. Multiple alignment was performed with LocARNA through the Freiburg RNA Tools webserver (http://rna.informatik.uni-freiburg.de) [27,28] with manual corrections as necessary.

### Sequence data and phylogenetic analyses

Several strategies were used to quantify and understand the distribution of polymorphisms in *Aylacostoma*. The number and nucleotide composition of haplotypes in *Aylacostoma* species were analysed in BioEdit 7.0.9 [29]. Nucleotide substitutions were examined in relation to conserved sequence motifs, alignment and secondary structure of the third domain of the 12S gene predicted for molluscs and other invertebrates. A secondary structure model was derived following the template proposed by Hickson et al. [23] and contrasted by comparison with structural diagrams for other molluscs (e.g. [30]). Genetic distances were estimated in MEGA 6.06 [31] using the number of differences (*p*) and the Kimura´s two-parameter (K2P) substitution model. Phylogenetic analyses were performed using neighbour-joining (NJ), maximum parsimony (MP), maximum likelihood (ML) and Bayesian inference (BI). The NJ analysis was conducted with MEGA 6.06 with the K2P model. The MP analysis was carried out using PAUP*4.0b10 [32] via the branch and bound algorithm (for smaller datasets) with gaps treated as missing and character states treated as unordered and equally weighted. For the ML analysis, the optimal model of nucleotide substitution (TrN+G) was selected with the likelihood-ratio test by means of the corrected Akaike Information Criterion (AICc) as implemented in jModelTest 2.1.7 [33]. The ML analysis was conducted in PhyML [34] via the Phylemon2 webserver (http://phylemon.bioinfo.cipf.es) [35]. Nodal support values were assessed by bootstrapping with 1000 (NJ, MP) and 100 (ML) replicates [36]. The BI was performed with MrBayes 3.2.6 [37] with the parameters from the best model (HKY+G) as identified by jModelTest under the Bayesian information criterion. Two runs were performed simultaneously with four Markov chains for 1000000 generations, sampling every 100 generations, with a burn-in of 10%.

### Species delimitation

Two approaches were used to explore species boundaries in the *Aylacostoma* dataset: the Automatic Barcode Gap Discovery (ABGD) method [38], and the *K*/θ method [39,40]. The ABGD is a non-tree-based distance method that has been used to define primary species hypotheses [41–44]. The aligned dataset (excluding outgroups) was analysed via the ABGD web-server (http://wwwabi.snv.jussieu.fr/public/abgd/), with default parameters using the K2P model. The *K*/θ method was conducted to assess the status of *Aylacostoma* species under the Evolutionary Genetic Species Concept (EGSC) [39,40]. This method, based on population genetic theory, requires a gene tree to identify putative sister clades and distance matrices to estimate genetic variation within and between clades [42]. It has recently been used to describe *A*. *brunneum* based on partial sequences of the COI gene, and was suggested by Vogler et al. [13] as a useful framework for comprehensively reviewing the status of the species in this genus. The *K*/θ method was performed following the procedures described in Schön et al. [45] and Birky [40]. In order to fulfil the *K*/θ criteria, sister clades must have *K*/θ ≥ 4 to be considered different species with probability ≥ 0.95 ([40] and references therein). Mean pairwise differences between clades were estimated in MEGA 6.06.

### Divergence time estimates

In order to estimate relative divergence times, the molecular clock hypothesis was tested in MEGA 6.06 using Tajimas´s non-parametric relative rate test [46], and by means of a likelihood-ratio test on ML trees with and without enforcing a molecular clock. Divergence time (*T*) was estimated following Vogler et al. [16] as *T* = *Da*/2μ, where *Da* is the net nucleotide divergence [47] and 2μ indicates the divergence rate (hereafter net divergence approach). Net divergence and standard errors (SE) were estimated in MEGA 6.06 under the K2P model with 1000 bootstrap replicates. 95% confidence intervals (CI) were calculated as ±1.96 SE of the net distances [48]. Divergence times were also estimated using a Bayesian approach implemented in BEAST 1.8.3 [49] under the HKY+G model (as identified by jModelTest under the Bayesian information criterion), assuming a strict clock and the Yule process to model speciation. The posterior distribution of divergence times was obtained by a MCMC sampling run of 100000000 generations, sampling every 1000 generations [50]. Convergence was confirmed using Tracer 1.6.0 [51,52]. Estimation of the time to the most recent common ancestors (TMRCAs), and associated 95% high posterior density (HPD) intervals were obtained after removing a burn-in of 10%. For both time estimation approaches, we used a divergence rate of 0.6% per million year based on Rumbak et al. [53], which has been widely used in phylogenetic and phylogeographic studies of gastropods (e.g. [54,55]).

## Results

### Sequence data and phylogenetic analyses

**Fig 1:**
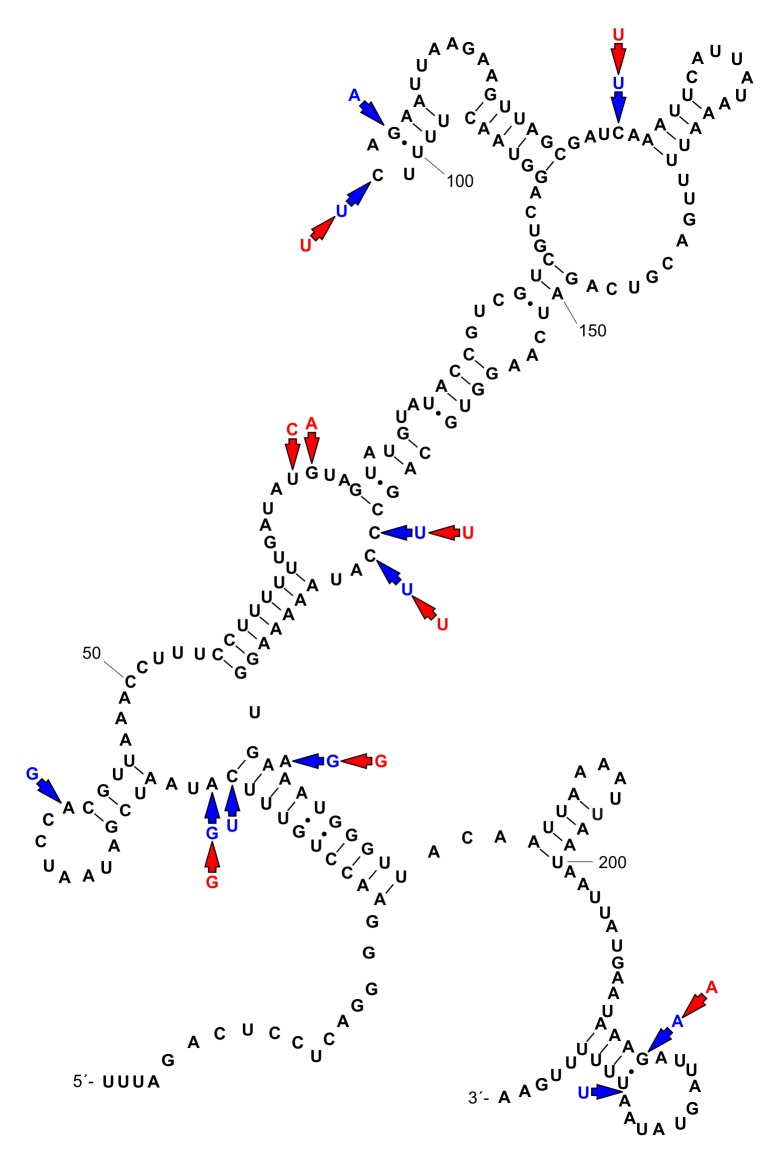
Secondary structure model of the third domain of the 12S mt rRNA gene for *Aylacostoma stigmaticum*. Mutational changes for *A*. *brunneum* and *A*. *chloroticum* are indicated by red and blue arrows, respectively (see Table 2).

Partial 12S sequences of *Aylacostoma* individuals from the High Paraná River consisted of 233 base pairs (bp) for *A*. *stigmaticum* and *A*. *brunneum*, and 234 bp for *A*. *chloroticum*. There were 13 variable positions in the sequence alignment (234 bp). Each species was characterized by a single haplotype. *Aylacostoma stigmaticum* differed from *A*. *brunneum* by nine nucleotides, and from *A*. *chloroticum* by ten nucleotides and one indel. *Aylacostoma brunneum* and *A*. *chloroticum* differed in five nucleotides and one indel (Table 2). Base frequencies, as well as AT and GC content are shown in Table 3. The secondary structure was conserved amongst the three species with ten polymorphic sites occurring in unpaired regions; the three remaining variable positions involved stems and represented alternative basepairing not affecting the secondary structure (Fig 1). Sequence divergences are presented in Table 4.

**Table 2:** Polymorphic positions based on a 234 bp fragment of the 12S mt rRNA gene for *Aylacostoma* species.

	27	28	42	67	68	102	104	123	163	164	177	214	225
*Aylacostoma stigmaticum*	C	A	A	T	G	C	G	C	C	C	A	G	–
*Aylacostoma brunneum*	∙	G	∙	C	A	T	∙	T	T	T	G	A	–
*Aylacostoma chloroticum*	T	G	G	∙	∙	T	A	T	T	T	G	A	T

Numbers indicate the position of variable sites. *Aylacostoma stigmaticum* is shown as reference sequence; dot indicates identity with the reference sequence, dash represent a gap.

**Table 3:** Nucleotide composition of 12S mt rRNA sequences for *Aylacostoma* species.

	*A*	*C*	*G*	*T*	*AT* content	*GC* content
*Aylacostoma stigmaticum*	81 (34.76%)	33 (14.17%)	39 (16.74%)	80 (34.33%)	69.10%	30.90%
*Aylacostoma brunneum*	81 (37.76%)	30 (12.88%)	39 (16.74%)	83 (35.62%)	70.39%	29.61%
*Aylacostoma chloroticum*	80 (34.19%)	28 (11.97%)	40 (17.09%)	86 (36.75%)	70.94%	29.06%

**Table 4:** Genetic distances between *Aylacostoma* species. Uncorrected (below the diagonal) and corrected (K2P; above the diagonal) distances are shown.

	*Aylacostoma stigmaticum*	*Aylacostoma brunneum*	*Aylacostoma chloroticum*
*Aylacostoma stigmaticum*	–	0.040200	0.044873
*Aylacostoma brunneum*	0.038627	–	0.021933
*Aylacostoma chloroticum*	0.042918	0.021459	–

**Fig 2:**
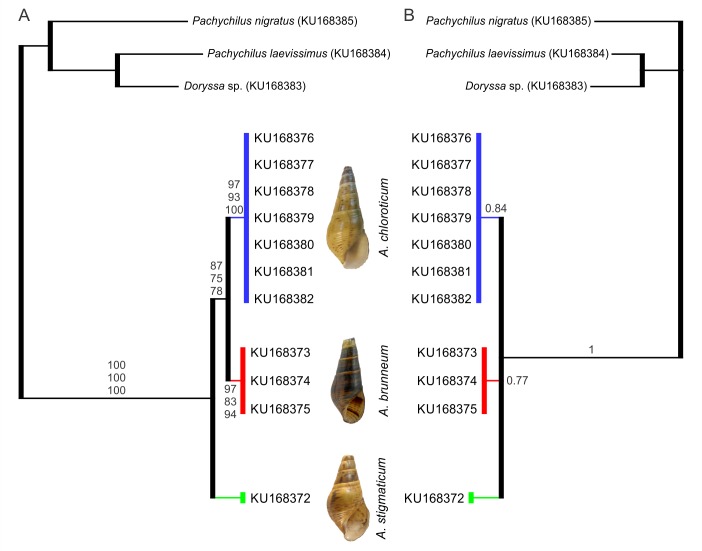
Phylogenetic trees of *Aylacostoma* species from the High Paraná River based on partial sequences of the of 12S mt rRNA gene. A, neighbour-joining tree. Bootstrap values for NJ, MP and ML trees, respectively, are shown above and below the branches. B, Bayesian consensus tree with posterior probabilities. Numbers within groups are GenBank accession numbers.

Results of the NJ, MP, ML and BI analyses all supported *Aylacostoma* as monophyletic with high bootstrap and posterior probability support (Fig 2). This group was further separated into three phylogenetic lineages, which represented *A*. *chloroticum*, *A*. *brunneum* and *A*. *stigmaticum*. In the results of the NJ, MP, and ML analyses, *A*. *chloroticum* and *A*. *brunneum* were supported as sister taxa, while in the results of the BI analysis, the relationships among species were unresolved.

### Species delimitation

**Fig 3:**
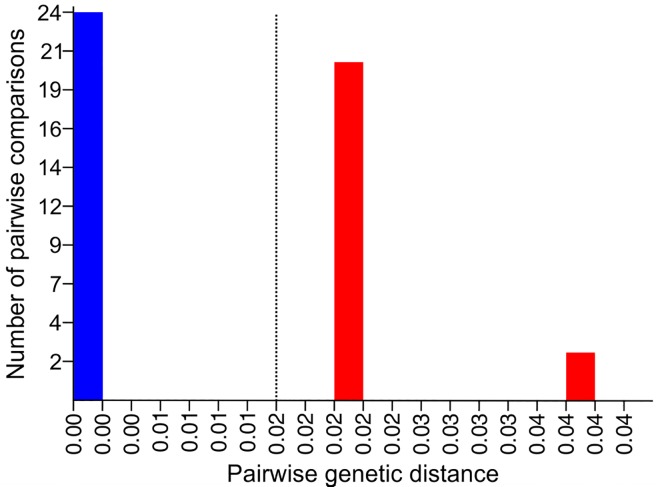
Frequency distribution of pairwise genetic distances for the 12S mt rRNA gene in the ABGD analysis. Pairwise distances were calculated using the K2P model. Dashed line corresponds to maximum value of intraspecific genetic divergences that resulted in stable candidate species.

The ABGD approach revealed a trimodal pairwise genetic distance (K2P) distribution (Fig 3) and resulted in seven sequence partitions that were stable across three groups (*A*. *chloroticum*, *A*. *brunneum*, and *A*. *stigmaticum*) at prior maximum intraspecific divergence values of 0.0215 and below (Table 5). The results of the *K*/θ method are shown in Table 6. All of the *K*/θ ratios were higher than 4 and the EGSC criteria were clearly fulfilled. Thus, *A*. *chloroticum*, *A*. *brunneum*, and *A*. *stigmaticum* were recognized as distinct evolutionary genetic species.

**Table 5:** Results of ABGD analysis for the 12S mt rRNA dataset using the K2P model to calculate pairwise distances.

Partition no.	Groups	*P*-value
1	3	0.001000
2	3	0.001668
3	3	0.002783
4	3	0.004642
5	3	0.007743
6	3	0.012915
7	3	0.021544

*P*, prior maximum intraspecific divergence.

**Table 6:** *K*/θ ratio calculations for *Aylacostoma* 12S mt rRNA sequences.

	θ	*K*	*K/*θ ratio	n1, n2
*A*. *chloroticum*–*A*. *brunneum*	0.0043165	0.0219333	5.08	7, 3
*A*. *brunneum–A*. *stigmaticum*	0.0043165	0.0402002	9.31	3, 1
*A*. *chloroticum–A*. *stigmaticum*	0.0014333	0.0448731	31.31	7, 1

θ, mean pairwise sequence difference within a clade; *K*, mean pairwise sequence difference between clades; n1, n2, number of sequences within each of the clades compared.

### Divergence time estimates

**Fig 4:**
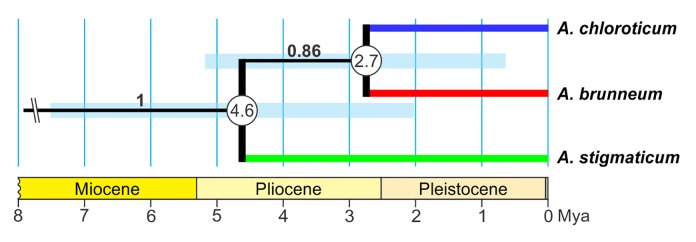
Divergence times for *Aylacostoma* species obtained from BEAST analysis of the 12S mt rRNA dataset. Numbers within circles indicate divergence times in millions of years. Bars at each node represent 95% confidence intervals. Posterior probabilities are shown on branches. Time axis (in millions of years) adapted from the International Chronostratigraphic Chart [56].

Tajima’s relative rate test and likelihood-ratio test indicated a clock like behaviour for the 12S dataset. Divergence between *A*. *stigmaticum* and the common ancestor of *A*. *chloroticum* and *A*. *brunneum* is inferred to have occurred roughly 6.57 (net divergence)– 4.64 (Bayesian) million years ago (mya), between the late Miocene and mid-Pliocene (Fig 4, Table 7). The maximum time interval for this divergence is estimated to be 10.63–2.04 mya (Table 7 and S1 Table). Subsequently, *A*. *brunneum* and *A*. *chloroticum* are estimated to have split between 3.75 (net divergence)– 2.71 (Bayesian) mya, between the mid-Pliocene and early Pleistocene (Fig 4, Table 7). The maximum time interval inferred for this split is estimated to be 0.54–6.97 mya (Table 7 and S1 Table).

**Table 7:** Divergence times among *Aylacostoma* species based on net divergence and Bayesian approaches.

	Net divergence approach		Bayesian approach	
Node	*T* = *Da*/2μ	95% CI	Mean	95% HPD
*A*. *stigmaticum / A*. *chloroticum*–*A*. *brunneum*	6.57	2.50–10.63	4.64	2.04–7.56
*A*. *chloroticum / A*. *brunneum*	3.75	0.54–6.97	2.71	0.67–5.21

*T*, divergence time; *Da*, net nucleotide divergence; 2μ, divergence rate; CI, confidence interval; HPD, highest posterior density interval.

## Discussion

Material deposited in natural history collections represent a valuable, but limited, resource for providing critical anatomical, morphological, ecological and genetic information on extinct species [57,58]. Historical DNA has enabled ecological, evolutionary, taxonomic and conservation studies, including for example, the identification of “extinct species” in captivity misassigned to extant taxa, refinement of evolutionary relationships, as well as the identification of cryptic diversity [59–61].

As for most invertebrates, genomic quality tissues are not usually available in museum collections, as most specimens were not preserved for this purpose [58,59]. Acquiring genetic information from shells is possible but has remained challenging as protocols for extracting DNA from empty shells have met with limited success, and in some cases requires complete dissolution of the shells [58,62]. In the present study, dissolution of shells of the extinct *A*. *stigmaticum* was not possible, since the only material available for study was a name-bearing type. Consequently, DNA was obtained here in a similar way as described by Caldeira et al. [63] by means of an extensive incubation period, after which the shell was recovered and returned to the MLP collection. From such DNA, it was possible to amplify and generate partial sequences of the 12S gene, although we failed to amplify the COI and cyt *b* regions. These results are in agreement with expectations for DNA obtained from historical material which is expected to be highly degraded, and PCR amplification generally restricted to short amplicons (<200 bp) [58,59]. Amplification of the standard COI barcoding region in molluscs has proven to be particularly problematic or impossible with historical material using standard primers [64].

Although failure to amplify the COI and cyt *b* genes for *A*. *stigmaticum* prevent us from making comparisons with available sequence data for *Aylacostoma*, comparisons of 12S sequences yielded results almost identical to those found for COI and cyt *b* by Vogler et al. [13] in terms of molecular identification while showing lower interspecies variability (ca. 2.1% for 12S vs 4.41% for COI and 6.65% for cyt *b*) reflecting its slower substitution rate [65,66]. Not unexpectedly, then, 12S did not capture the haplotype clade diversity in *A*. *chloroticum* previously characterized with the COI gene [13,16]; consequently, this marker appears unsuitable for exploring intraspecific variation in these species.

Consistent with the findings of Lydeard et al. [67,68] and Ramírez & Ramírez [69] for the mitochondrial LSU rRNA gene (16S) of molluscs, the secondary structure model derived here demonstrated that most of the sequence variation among the *Aylacostoma* species was allocated in loops. As this is the first such structural model for *Aylacostoma*, comparisons with other congeners are presently not possible, although our results suggest that the 12S region and the secondary structure model based on the extinct *A*. *stigmaticum* have considerable potential for further comparative analyses of structural changes within the genus and other molluscan taxa.

Despite the apparent lack of utility of the 12S gene to assess intraspecific variability in *Aylacostoma*, our findings support this region as an efficient marker for the discrimination of species. This was demonstrated herein by means of two species delimitation approaches, where three genetic lineages were identified by the ABGD method, all of which fulfilled the criteria of the Evolutionary Genetic Species Concept. Consequently, this confirms *A*. *stigmaticum* as a distinct evolutionary genetic species, and the previous treatment of *A*. *chloroticum* and *A*. *brunneum* as evolutionary genetic species based on COI data [13]. Interestingly, the validity of the extinct *A*. *stigmaticum* was questioned by de Castellanos [21] on the basis of shared features of the shell with the other species inhibiting the High Paraná region. The original description of *A*. *stigmaticum* was based solely on shell features: a conical to ovate shell, yellow horn colour, low spire, last whorl somewhat convex; surface almost smooth, last whorl sculptured by low spiral cords, a spiral band of reddish spots, and the presence of irregular black spots on the surface of all specimens, with the latter being the most conspicuous feature [15,18]. Until now, apart from the fewer number of whorls, its relatively low spire and the inflation of the body whorl, the distinctive coloration pattern represents the most notable distinguishing feature of *A*. *stigmaticum*, whereas *A*. *chloroticum* is characterized by greenish-yellow to greenish-brown shells, with some specimens bearing minute, dark reddish brown, spiral spots, while *A*. *brunneum* has dark brown shells with alternating lighter bands, delicately decorated with minute, dark reddish brown, regularly spaced spiral spots [13]. Moreover, the present results complement those of Vogler et al. [70] who conducted a geometric morphometric analysis of available museum material for *A*. *guaraniticum*, *A*. *chloroticum* and *A*. *stigmaticum* documenting significant differences in shell shape, in which *A*. *stigmaticum* was found to possess a comparatively more globose shell and more ovate aperture. However, in that study affinities could not be assessed based only on overall similarity of the shell, as convergence in shell shape and ecophenotypic variation could not be rejected as confounding factors. Here, with the exception of the results of the BI analysis which were unresolved, phylogenetic analyses consistently supported a sister group relationship between *A*. *chloroticum* and *A*. *brunneum*, with the extinct *A*. *stigmaticum* grouping outside. It is important to note that the sequences of *A*. *chloroticum* presented an indel, treated as “missing” in the phylogenetic analyses, which generated a somewhat overestimated similarity among the species. DNA sequences for the extinct *A*. *guaraniticum* are still unavailable and further research is required to understand the evolutionary framework shaping diversification of *Aylacostoma* in the High Paraná River. However, by placing the present results into the context of previous research, the extinct *A*. *stigmaticum* provides interesting new insights into several aspects of the evolution of the High Paraná River species.

The Paraná River, the second largest of South American rivers, becomes the High Paraná River from the former Guairá Falls along the Brazil–Paraguay border, now flooded by the Itaipú Reservoir, which comprised a series of imposing falls with walls 100 m high [71]. Downstream, the river runs along the Argentina–Paraguay border along a fault line across a broad basaltic plateau known as Alto Paraná Encajonado [72], and continues without major changes until near 56°W, where it turns westward. From this point and before the impoundment of the Yacyretá Reservoir, the course historically formed a large and complex system of extensive anastomosing meanders and more than 300 islands, including many deeper passages and rapids in a floodplain 25–30 km wide extending to the Yacyretá–Apipé rapids, now the site of the Yacyretá dam [9,71,73,74]. This unique stretch of river has been considered as a biogeographic crossroads by Arzamendia & Giraudo [75] and is currently recognized as a transition zone between the Atlantic Forest and Chaco biogeographical provinces. Although the origins of its endemic molluscan fauna have been little investigated, bearing in mind that all *Aylacostoma* species from Argentina–Paraguay have been described from such a particular stretch of the river within a narrow distribution range, the perception of *A*. *stigmaticum* as a distinct, endemic species, further highlights that the now flooded convergence zone of the High Paraná River and the Alto Paraná Encajonado was a micro-hotspot supporting a highly endemic molluscan fauna [76,77].

Geomorphological history has been implicated in playing a major role in the evolution of *Aylacostoma* species in the High Paraná River [9,10,16]. In this context, divergence times obtained with 12S for the split between *A*. *chloroticum* and *A*. *brunneum* yielded an equivalent time estimate to that reported by Vogler et al. [16] using the COI gene, and suggest a divergence between the mid-Pliocene and early Pleistocene. The split between *A*. *stigmaticum* and the common ancestor of *A*. *chloroticum* and *A*. *brunneum* likely occurred roughly 6.57–4.64 mya between the late Miocene and mid-Pliocene. These divergence estimates are consistent with known episodes in the geomorphological history of the High Paraná River, which may have driven the intra-basin diversification of *Aylacostoma*. During the middle and late Miocene, the “Paranense Sea” is recognized as one of the most widespread Atlantic marine transgressions that covered parts of Argentina, Uruguay, and southern portions of Brazil, Bolivia and Paraguay [78–80]. Miocene marine transgressions are considered to have played a significant role in species origin, diversification and distribution patterns of South America diversity, particularly among its freshwater fauna [81–84]. Geological evidence supports two cycles of marine transgression into the Paraná basin in the late Miocene, the first between 15–13 mya and the second between 10–5 mya [78]. The latter coincides with our estimates for the split between *A*. *stigmaticum* and the common ancestor of *A*. *chloroticum* and *A*. *brunneum*. Consequently, as for the fish of the Paraná River [84], it can be hypothesized that marine incursion into the Paraná basin fragmented the range of a continuously distributed ancestor promoting speciation. On the other hand, as has been suggested by Vogler et al. [16], the more recent divergence between *A*. *chloroticum* and *A*. *brunneum* may have occurred in a large and heterogeneous alluvial fan complex of the Paleoparaná [85] which formed after the withdrawal of the “Paranense Sea”, through the colonization of transitional environments, in which geographic fragmentation and ecological specialization promoted differentiation. Thus, *Aylacostoma* snails from the High Paraná River may represent a species flock as the result of a geographically localized adaptive radiation during the late Miocene and Pliocene. However, as stated by Vogler et al. [16] further paleontological studies are required to understand the likely complex interplay between spatial, biological and ecological factors driving speciation of *Aylacostoma* in this region [86].

Finally, the data presented herein establish an evolutionary framework for future comparative analyses of *Aylacostoma* and other South American thiarids, in which most of the species are known primarily from shells in natural history collections. Our results reveal that DNA can be obtained from dried specimens of freshwater species at least 80 years after their collection, similar to previous findings in land snails. As demonstrated here historical DNA can provide invaluable information for elucidating evolutionary patterns and processes, and we suggest this resource be more frequently incorporated into the malacological toolbox for further studies aimed at reconstructing the tempo and mode of evolution in gastropods.

## Supporting Information

S1 Table Estimate of divergence times among *Aylacostoma* species from the High Paraná River using the net divergence approach under the K2P model. See text for details.
(DOCX)
