# Supplementary material for: Insights into the Evolutionary History of an Extinct South American Freshwater Snail Based on Historical DNA
Source: PLoS One. 2016 Dec 29;11(12):e0169191. doi: 10.1371/journal.pone.0169191 (PMC5199097; doi:10.1371/journal.pone.0169191)
Supplement: S1 Table — See text for details. (DOCX) [file pone.0169191.s001.docx]

**S1 Table. Estimate of divergence times among *Aylacostoma* species from the High Paraná River using the net divergence approach under the K2P model.** See text for details.

|  | ***A. stigmaticum* / *A. chloroticum* and *A. brunneum***  Rate: 0.6% per My | ***A. chloroticum* / *A. brunneum***  Rate: 0.6% per My |
| --- | --- | --- |
| *Da* | 0.0394171491766571 | 0.0225263319704716 |
| SE | 0.0124399635914037 | 0.0098389491214427 |
| CI (*Da* ± 1.96 SE) | 0.015034821 – 0.063799478 | 0.003241992 – 0.041810672 |
| 2μ | 0.006 | 0.006 |
| *T* | **6.569524863** | **3.754388662** |
| CI (*T*) | Min: 2.505803423  Max: 10.6332463 | Min: 0.540331949  Max: 6.968445375 |
| **ETR** | **2.51 – 10.63 My** | **0.54 – 6.97 My** |
|  |  |  |

*Da*, net nucleotide divergence; SE, standard error; CI, 95% confidence interval; 2μ, divergence rate; *T*, divergence time; My: million years; ETR, estimated time range. The divergence time was estimated as *T* = *Da*/2μ.
